# Attenuation of ROS/Chloride Efflux-Mediated NLRP3 Inflammasome Activation Contributes to Alleviation of Diabetic Cardiomyopathy in Rats after Sleeve Gastrectomy

**DOI:** 10.1155/2022/4608914

**Published:** 2022-04-19

**Authors:** Songhan Li, Shuohui Dong, Bowen Shi, Qian Xu, Linchuan Li, Shuo Wang, Wenjie Zhang, Mingwei Zhong, Jiankang Zhu, Yugang Cheng, Guangyong Zhang, Sanyuan Hu

**Affiliations:** ^1^Department of General Surgery, Shandong Qianfoshan Hospital, Cheeloo College of Medicine, Shandong University, Jinan, Shandong 250014, China; ^2^Department of General Surgery, The First Affiliated Hospital of Shandong First Medical University, Jinan, Shandong 250014, China; ^3^Department of Gastroenterological Surgery, Peking University People's Hospital, Beijing 100044, China

## Abstract

Diabetic cardiomyopathy (DCM) can develop in diabetes mellitus and is a major cause of morbidity and mortality. Surgical bariatric surgery procedures, such as sleeve gastrectomy (SG), result in remission of type 2 diabetes and have benefits regarding systolic and diastolic myocardial function. The NLR family pyrin domain containing 3 (NLRP3) inflammasome appears to participate in the development of DCM. However, whether SG surgery affects myocardial NLRP3 inflammasome-related pyroptosis to improve cardiac function remains unclear. This study was aimed at investigating the effect of SG surgery on NLRP3-associated pyroptosis in rats with DCM. We also examined cellular phenotypes and molecular mechanisms in high glucose-stimulated myocytes. The rat model of DCM was established by high-fat diet feeding and low-dose streptozotocin injection. We observed a metabolic benefit of SG, including a reduced body weight, food intake, and blood glucose levels and restored glucose tolerance and insulin sensitivity postoperatively. We observed a marked decline in glucose uptake in rats with DCM, and this was restored after SG. Also, SG alleviated the dysfunction of myocardial contraction and diastole, delayed the progression of DCM, and reduced the NLRP3 inflammasome-mediated myocardial pyroptosis *in vivo*. H9c2 cardiomyocytes showed membrane disruption and DNA damage under a high glucose stimulus, which suggested myocardial pyroptosis. Using a ROS scavenger or chloride channel blocker *in vitro* restored myocardial NLRP3-mediated pyroptosis. Furthermore, we found that chloride efflux acted downstream of ROS generation. In conclusion, SG may ameliorate or even reverse the progression of DCM. Our study provides evidence that the SG operation alleviates NLRP3 inflammasome dysregulation in DCM. Clearance of ROS overburden and suppression of chloride efflux due to SG might act as the proximal event before inhibition of NLRP3 inflammasome in the myocardium, thus contributing to morphological and functional alleviation of DCM.

## 1. Introduction

Diabetic cardiomyopathy (DCM) is a distinct disease entity that develops in diabetes mellitus (DM) and does not come after any coronary artery disease, hypertension, or valvular heart disease [1, 2]. Currently, DCM is recognized as the predominant cause of morbidity and mortality among people suffering from DM [3]. At the onset of diabetes, owing to insulin resistance and metabolic shifts in cardiomyocytes, intracellular lipid and lipotoxicity were accumulated; afterwards, mitochondrial dysfunction and accumulation of reactive oxygen species (ROS) occurred in cardiomyocytes [2]. Ultimately, these pathological alternations bring about cardiomyocyte death, inflammatory response, and fibrotic remodeling [4].

Surgical bariatric surgery procedures such as sleeve gastrectomy (SG) are currently one of the most prevalent treatments for obesity and its related metabolic disorders [5]. Plenty of evidence have shown that SG results in effective remission of type 2 diabetes (T2DM) and associated complications [6, 7]. A couple of studies evaluated that the SG had benefits regarding systolic and diastolic myocardial function in patients with DCM or in rat models [8, 9], whereas, to date, the underlying mechanism of SG remains to be integrated.

Pyroptosis is a type of proinflammatory programmed cell death [10]. Activation of the canonical NLR family pyrin domain containing 3 (NLRP3) inflammasome can engage caspase-1 for cleaving gasdermin D (GSDMD) as well as turning proforms of interleukin- (IL-) 1*β* and IL-18 into active forms, finally leading to cell membrane pore formation and release of inflammatory cytokines [11]. A previous study showed that a deficit of NLRP3 improved insulin sensitivity in obese mice, which suggests that NLRP3 plays a significant role in metabolism [12]. Previous studies also showed that ablation of NLRP3 in rats with DCM could significantly reduce myocardial pyroptosis and induce inflammatory response [13, 14]. These previous findings suggest that the NLRP3 inflammasome participates in the development of DCM. Additionally, studies have shown that bariatric surgery inhibits NLRP3 activation in pancreatic islets, hepatocytes, and adipose tissues, causing anticell death and anti-inflammatory effects [15–18]. Notwithstanding, whether SG surgery affects myocardial NLRP3 inflammasome-related pyroptosis for the improvement of cardiac function remains unclear.

To address these issues, we established a rat model of DCM by high-fat diet (HFD) feeding and low-dose streptozotocin (STZ) injection compared with chow diet-fed rats as controls. SG or sham surgery was performed to investigate the effect of SG surgery on NLRP3-associated pyroptosis in rats with DCM. Furthermore, cellular phenotypes and molecular mechanisms were examined in high glucose-stimulated myocytes.

## 2. Materials and Methods

### 2.1. Animals

All animal protocols were approved by the Medical Ethical Committee of the Shandong Provincial Qianfoshan Hospital, Shandong University. Male Wistar rats at the age of approximately 6 weeks were purchased from Beijing Weitong Lihua Experimental Animal Technology and were placed in an environment with access to feed and water with alternate 12-hour light and dark cycles at 22 ± 2°C. After one-week-long adaptive feeding, two groups of rats were kept in a high-fat diet (HFD, 60% of calories as fat, Xietong Biotech, Nanjing, China) for four weeks to induce obesity and insulin resistance, while the other group was fed with normal chow. After 12 h fasting, we intraperitoneally injected rats with streptozotocin (STZ; 35 mg/kg, Sigma-Aldrich, St. Louis, MO, USA) dissolved in sodium citrate buffer pH 4.5 (Solarbio, Beijing, China) to produce the type 2 diabetic models. One week after STZ administration, fasting blood glucose (FBG) was measured with a glucometer (Roche One Touch Ultra, LifeScan, CA, USA). Only rats with FBG exceeding 11.1 mmol/L were regarded as promising diabetic models [19]. Diabetic rats were fed with HFD for 8 weeks after STZ injection for a stable DCM model establishment, as described by previous studies; after eight-week STZ injection when the stable T2DM model is established, the onset of cardiac dysfunction is observed as well [20, 21].

### 2.2. Surgical Procedures

Prior to surgery, rats were fed with 10% Ensure (Abbott Laboratories, Abbott Park, IL, USA) for 2 days and then fasted overnight and underwent SG or sham surgery under anesthesia with 2% isoflurane. SG and sham surgery were performed in accordance with a previous report [22]. Afterwards, the rats were caged individually, and the diet was transited gradually from liquid diet to high-fat diet consistent with the preoperative feeding strategy. Food intake and body weight were recorded per week. The rats were euthanized 8 weeks after surgery.

### 2.3. Oral Glucose Tolerance Test and Insulin Tolerance Test

An oral glucose tolerance test (OGTT) and insulin tolerance test (ITT) were performed before surgery and at 8 weeks after surgery. Blood glucose was monitored from the tail vein of fasting rats at baseline and 10, 30, 60, and 120 min after the administration of intragastric gavage of 20% glucose (1 g/kg) for the OGTT or intraperitoneal injection of human insulin (0.5 IU/kg) for the ITT. The homeostasis model assessment of basal insulin resistance (HOMA-IR) was evaluated as FBG (mmol/L) × fasting insulin (mIU/L)/22.5.

### 2.4. Cardiac Function

Echocardiography examination was performed before the surgery to prove the successful establishment of DCM models and at 8 weeks after surgery to observe the clinical effects of SG. Rats were anesthetized with 2% isoflurane for transthoracic echocardiography with an RMB710 transducer using the Vevo 3100 system (VisualSonics, Toronto, Canada). The left ventricular end-diastolic diameter (LVEDd), left ventricular ejection fractions (LVEF), fractional shortening (FS), and early-to-late mitral diastolic flow ratio (E/A) along with isovolumic relaxation time (IVRT) were measured.

### 2.5. ^18^F-FDG PET of Rat Heart Imaging

PET scans were performed at 8 weeks after surgery. Fasting rats were made to inhale isoflurane and fixed with cannulas. After intravenous injection with 29.6 MBq (800 *μ*Ci) of ^18^F-FDG, rats were maintained for one hour and the hearts were scanned with a Positron Emission Computed Tomography (PET) scanner (Metis 1800, Madic Technology, China).

### 2.6. Histology and Immunohistochemistry

Each rat myocardial sample, formalin-fixed and paraffin-embedded, was cut into 5 *μ*m sections. Slides were stained with haematoxylin and eosin (H&E) for standard histology. The distribution of collagen was carried out by Masson's trichrome staining and picrosirius red staining. The quantitative analysis of the cardiomyocyte size and collagen volume was measured with the Image-Pro Plus 6.0 (Media Cybernetics, Bethesda, MD, USA). Frozen sections were stained with Oil Red O to detect liquid content. Terminal deoxynucleotidyl transferase-mediated dUTP nick end-labeling (TUNEL) staining was utilized to assess cell death (Servicebio, Wuhan, China). After antigen retrieval in a citrate buffer (0.01 M, pH 6.0) and endogenous peroxidase blocking, slides were incubated with primary antibodies against collagen I (Affinity, AF0134, 1 : 100), collagen III (Affinity, AF0136, 1 : 100), NLRP3 (Abcam, ab210491, 1 : 200), ASC (Affinity, DF6304, 1 : 100), GSDMD (Affinity, AF4012, 1 : 100), IL-1*β* (Abcam, ab9722, 1 : 200), IL-18 (ProteinTech, 10663-1-AP, 1 : 300), and NEK7 (Affinity, DF4467, 1 : 100) at 4°C overnight. Thereafter, the tissues were incubated with HRP-conjugated secondary antibodies at 37°C for 30 minutes and observed with microscope (ZEISS, Axio Vert.A1, Jena, Germany).

### 2.7. Transmission Electron Microscopy

One-millimeter LV cubes were prefixed in 2.5% glutaraldehyde overnight at 4°C and postfixed in 1% osmium tetroxide for 2 h at 4°C. Samples were then dehydrated through an ethanol series and embedded in Epon. After complete polymerization and cutting into ultrathin sections, the sections were stained with uranyl acetate and lead citrate which were then examined using a transmission electron microscope (Hitachi, HT-7800, Japan).

### 2.8. Cell Culture and Treatment

H9c2 rat cardiomyocytes were cultured in low glucose DMEM (5.5 mM) supplemented with 10% FBS in a 5% CO_2_ thermostatic (37°C) incubator. After serum starvation overnight, cells were exposed to high glucose (HG, 33.3 mM) for 48 h. For the ROS scavenger, H9c2 cells were pretreated with 1 mM N-acetyl-L-cysteine (NAC, MCE, Shanghai, China) before the stimulation of HG for 12 h. For chloride efflux inhibition, additionally, 100 *μ*M indanyloxyacetic acid-94 (IAA-94, MCE, Shanghai, China) was administered for 24 h.

### 2.9. Flow Cytometry

The levels of glucose uptake, intracellular ROS load, and chloride concentration were determined through flow cytometry. When indicated H9c2 cells in 6-well plates had reached 80% confluence, they were treated under respective stimulation as described in Section 2.8. After being washed with cold PBS three times, 2-deoxy-glucose analog (2-NBDG, MCE, Shanghai, China) was added at a final concentration of 50 *μ*M in glucose-free DMEM for 30 min. And H9c2 cells ware incubated with 2 *μ*M DCFH-DA (Beyotime, Shanghai, China) in low glucose DMEM for 20 min. Also, 10 *μ*M N-(ethoxycarbonylmethyl)-6-methoxyquinolinium bromide (MQAE, MCE, New Jersey, USA) was added into the complete low glucose DMEM, and cells were incubated for 60 min. Cells were all cultured with probes in a 37°C 10% CO_2_ humidified incubator. Then, the cells were washed with ice-cold PBS and digested with trypsin-EDTA and kept on ice protected from light. The fluorescence intensity of 2-NBDG, DCFH-DA, and MQAE (FITC channel) in labelled cells was detected through a BD FACSAria II instrument (BD, USA). 20,000 events from each specimen were measured to pledge sufficient data. The data were analyzed by FlowJo v10 (FlowJo, Ashland, OR, USA), and the polygonal gating strategy was used to exclude debris.

### 2.10. Caspase-1 Activity Detection

The caspase-1 activity was detected with a Caspase-1 Activity Assay Kit (BestBio, Shanghai, China). Firstly, cells were lysed in 100 *μ*L lysis buffer on ice for 15 min. 10 *μ*L supernatant was retained and remixed with 90 *μ*L detection buffer and 10 *μ*L caspase-1 substrate (Ac-YVAD-MCA) at 37°C for 2 h. Then, the optical density of samples was read on a microplate reader (Bio-Rad, CA, USA) at 405 nm.

### 2.11. LDH Release Detection

Lactate dehydrogenase (LDH) in the supernatant was measured using the LDH Release Assay Kit (Beyotime, Beijing, China) according to the manufacturer's protocol. The percentage of LDH release was computed as (experimental LDH − spontaneous LDH)/(maximum LDH release − spontaneous LDH).

### 2.12. Glucose Determination in Supernatants

The content of glucose in the supernatants was quantified using a Glucose Colorimetric Assay Kit (GOD-POD Method, Elabscience, Wuhan, China). H9c2 cells were seeded into 6-well plates and grown to 80% confluency under respective stimulation. The culture medium was changed to low glucose DMEM after 48 h and incubated for 24 h. The enzyme solution was configured according to the instruction manual. 3 *μ*L of supernatant of all samples and 300 *μ*L enzyme solution was transferred to a 96-well plate. After incubation at 37°C for 15 min, the final OD values were determined at 505 nm in a spectrophotometer. The concentrations of glucose were recorded according to the standard curve.

### 2.13. Chloride Detection in Supernatants

The chloride concentration in the supernatants was examined using a Chloride Colorimetric Assay Kit (Elabscience, Wuhan, China). H9c2 cells were also seeded into 6-well plates with a distinctive stimulus. The culture medium was removed, and cells were incubated with low glucose DMEM for 24 h. The chromogenic agent was configured according to the standard protocol. 10 *μ*L of supernatants and 250 *μ*L of a chromogenic agent were added to a 96-well plate. The OD values were calculated at 460 nm after incubation at room temperature for 5 min. And concentrations of the samples were determined from the standard curve.

### 2.14. Cell Death Assay

The TUNEL assay was used to detect DNA fragmentation, and the probe E42 staining was used to evaluate the membranal disruption. Cells were cultured on cell climbing slices. The TUNEL assay was performed with a TUNEL Cell Apoptosis Detection Kit (Servicebio, Wuhan, China) according to the given protocol. The cellular membrane-disrupted cells were labeled probe E42 under the instruction of the Cell Membrane Integrity Assay Kit (BestBio, Shanghai, China). Nuclei were stained with DAPI. Images were acquired on a confocal microscope (Leica, TSP2, Wetzlar, Germany).

### 2.15. Western Blot

Heart tissue proteins were extracted with the Total Protein Extraction Kit for Muscles (Invent Biotechnologies, Beijing, China). Cell proteins were lysed in a RIPA lysis buffer (Solarbio, Beijing, China). Membrane protein of H9c2 cells were prepared using the Minute Plasma Membrane Protein Isolation Kit (Invent Biotechnologies, Beijing, China). The protein extracts were separated using the 10% SDS-PAGE and followed by transferring to the PVDF membrane (Millipore, Cork, Ireland). After one-hour blocking in 5% fat-free milk, the membrane was incubated with primary antibodies at 4°C overnight. At the following day, secondary antibody (ProteinTech, Wuhan, China, SA00001-1/2, 1 : 5000) incubation was performed before the enhanced chemiluminescence (Millipore, Billerica, USA) with imagination via an Amersham Imager 680 (GE, Boston, USA). We used antibodies against NLRP3 (Abcam, Cambridge, UK, ab210491, 1 : 1000), IL-1*β* (ab9722, 1 : 2000), procaspase-1+p10+p12 (ab179515, 1 : 1000), SOD2 (ab68155, 1 : 1000), IL-18 (ProteinTech, Wuhan, China, 10663-1-AP, 1 : 2000), TXNIP (18243-1-AP, 1 : 1000), CLIC1 (14545-1-AP, 1 : 1000), CLIC4 (66343-1-Ig, 1 : 2000), beta-tubulin (10068-1-AP, 1 : 5000), ATP1A1 (14418-1-AP, 1 : 10000), GSDMD (Affinity, Cincinnati, OH, USA, AF4012, 1 : 1000), ASC (DF6304, 1 : 1000), NEK7 (DF4467, 1 : 1000), NF-*κ*B p65 (CST, MA, USA, #8242, 1 : 1000), and phospho-NF-*κ*B p65 (Ser536, #3033, 1 : 1000).

### 2.16. RNA Extraction and Real-Time PCR

Total RNA of heart tissues and cell samples were extracted using a TRIzol reagent (Invitrogen, Carlsbad, CA, USA). cDNA synthesis was performed with a ReverTra Ace qPCR RT Kit (Toyobo, Osaka, Japan), and real-time PCR was performed using a SYBR Green Kit (Toyobo, Osaka, Japan) on the Roche LightCycler 480 System (Roche, Basel, Switzerland). The primer sequences are shown as in Table 1.

### 2.17. Immunofluorescence

The double-labeling localization of NLRP3 and CD68 were observed using immunofluorescence methods. 8 *μ*m thick frozen sections were blocked with 2% BSA and incubated with primary antibodies against NLRP3 (Abcam, ab210491) and CD68 (Abcam, ab955) overnight at 4°C. After incubation with two different fluorescent secondary antibodies for 30 minutes at 37°C, slides were stained with DAPI.

The expression and localization of protein CLIC1 and CLIC4 were observed via immunofluorescence. H9c2 cells were cultured in 24-well plates and fixed with 4% paraformaldehyde, after which 0.1% of Triton X-100 was used for membrane permeabilization and 2% BSA for blocking. Thereafter, cells were incubated with primary antibodies CLIC1 (ProteinTech, 14545-1-AP, 1 : 100) and CLIC4 (66343-1-Ig, 1 : 200) at 4°C overnight. After staining with fluorescent secondary antibody and DAPI, images were observed by an immunofluorescence microscopy (Olympus, IX73, Tokyo, Japan).

### 2.18. Statistical Analysis

The data were presented as mean ± standard deviation (SD) and were analyzed using SPSS 22.0 (SPSS, Chicago, USA). Body weight, food intake, and FBG over time were analyzed using the two-way ANOVA and Tukey-Kramer tests. Other data were analyzed among groups using one-way ANOVA with subsequent Bonferroni or Dunnett's T3 correction. *P* < 0.05 was considered statistically significant.

## 3. Results

### 3.1. Sleeve Gastrectomy Remarkably Alleviates General Characteristics and Glucose Homeostasis in Rats with Obesity and T2DM

**Figure 1:**
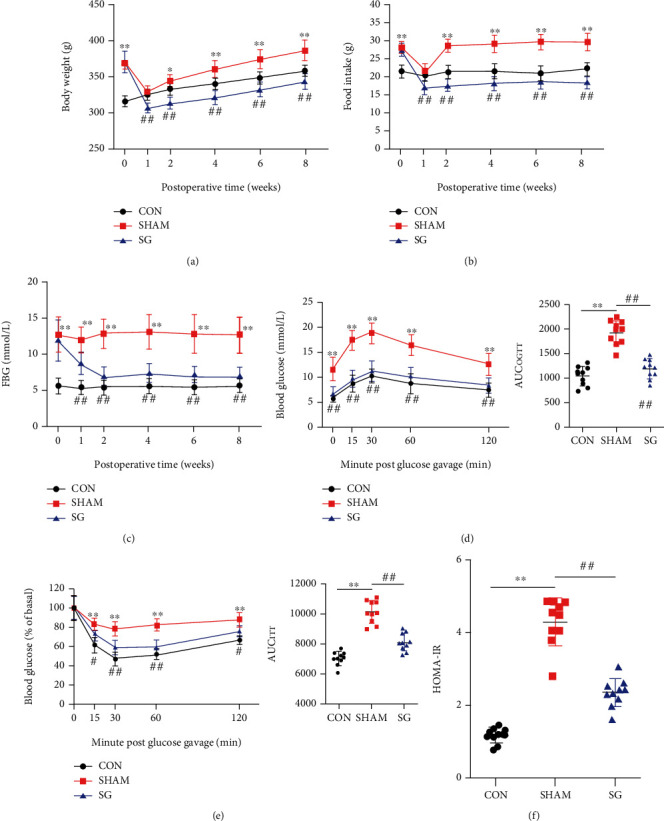
Effects of SG on body weight, food intake, and glucose homeostasis in diabetic rats. (a) Body weight, (b) food intake, and (c) fasting blood glucose before and after SG surgery. (d) OGTT curve and (e) ITT curve were carried out 4 weeks postoperatively. AUC_OGTT_ and AUC_ITT_ were calculated, respectively. (f) Values of HOMA-IR at 4 weeks after SG surgery. Data are presented as mean ± SD. ^∗^*P* < 0.05 and ^∗∗^*P* < 0.01 CON vs. SHAM; ^#^*P* < 0.05 and ^##^*P* < 0.01 SG vs. SHAM. *n* = 10 in each group.

Body weight curves of the three groups including the control group, sham group, and SG group are illustrated in Figure 1(a). Body weight in the sham surgery group and the SG group plateaued at 1 week postoperatively and gradually increased in weight thereafter. Body weight persistently showed a difference between the sham and SG groups. To determine the alternation in feeding behavior, we measured the food intake after surgery (Figure 1(b)). The sham and SG groups consumed fewer calories during the first week following surgery, which may have resulted from transient surgical stress. However, this appetite deficit of rats in the sham group was restored at a later time, while rats in the SG group remained hypophagic continuously, and this enabled maintenance of a reduced body weight. To evaluate the effects of SG on glucose homeostasis, FBG concentrations were regularly recorded (Figure 1(c)). FBG sharply decreased in the first 2 weeks and were stable afterwards. Rats in the SG group exhibited a substantial improvement in the ability to clear oral gavage of glucose, as reflected by a 38% drop in the area under the curve (AUC) relative to the sham group (Figure 1(d)). ITT was performed, and it showed a lower AUC in the SG group compared with the sham group, indicating that SG improved insulin sensitivity (Figure 1(e)). The HOMA-IR value significantly alleviated after SG, which was in agreement with the above-mentioned results (Figure 1(f)).

### 3.2. SG Attenuates Diabetic-Induced Cardiac Dysfunction and Myocardial Metabolism

**Figure 2:**
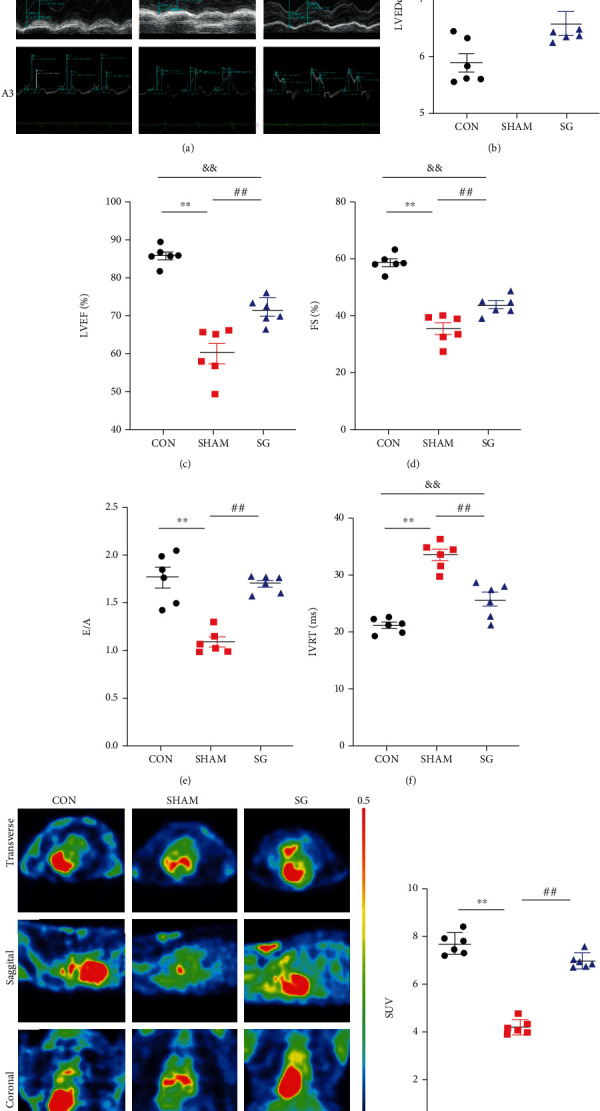
SG abrogates cardiac dysfunction and improves myocardial metabolism in rats with DCM. (a) Echocardiography: (a1) representative images of 2D echocardiograms; (a2) M-mode echocardiograms; (a3) mitral inflow shown by pulse-wave Doppler echocardiograms. Echocardiographic measurements including (b) LVEDd, (c) LVEF, (d) FS, (e) ratio of E/A, and (f) IVRT. (g) Representative transverse, sagittal, and coronal axis images of the heart showing ^18^F-FDG distribution via PET-scans. (h) Standard uptake value (SUV). Data are presented as mean ± SD. ^∗∗^*P* < 0.01 CON vs. SHAM; ^##^*P* < 0.01 SG vs. SHAM; ^&&^*P* < 0.01 CON vs. SG. *n* = 6 in each group.

An echocardiographic examination was carried out 8 weeks postsurgery for assessing cardiac function (Figure 2(a)). Rats that underwent sham surgery had a higher LVEDd and IVRT along with greater impaired LVEF and FS and a higher E/A ratio compared with those in the control group. Echocardiographic analysis demonstrated systolic and diastolic dysfunction in the sham group. However, in comparison to the sham group, SG surgery mitigated these unfavorable changes, as evidenced by significantly reduced LVEF and IVRT with a restored LVEF, FS, and E/A ratio (Figures 2(b)–2(f)).

Insulin resistance is one of the main triggers of DCM, of which impaired myocardial glucose uptake is a pathophysiological hallmark. PET scans were carried out after ^18^F-FDG injection to scale the levels of myocardial glucose uptake, and the standard uptake value (SUV) of the sham group was definitely decreased compared with the control group, yet SUV of the SG group was restored, indicating that SG surgery alleviated diabetic-induced myocardial insulin resistance (Figures 2(g) and 2(h)).

### 3.3. SG Reverses Myocardial Remodeling in Rats with DCM

**Figure 3:**
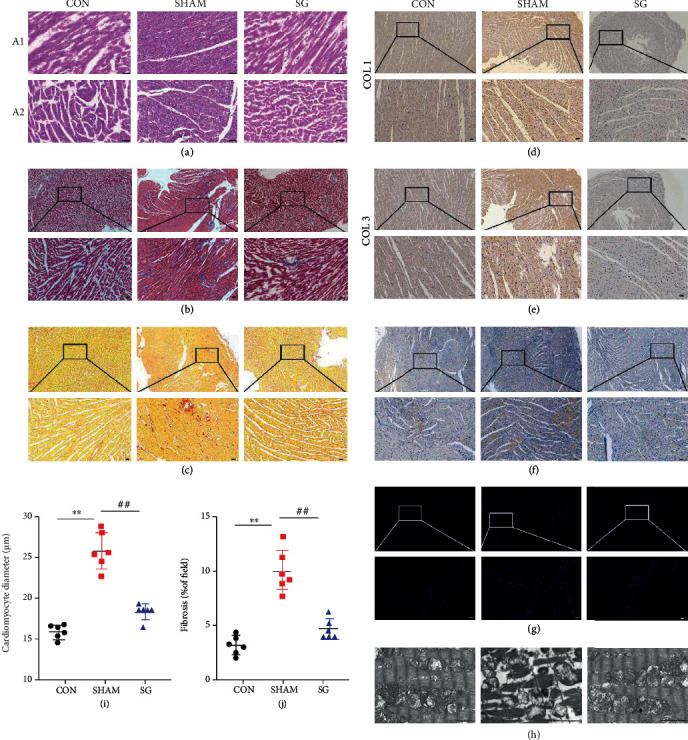
Effects of SG on diabetic-induced cardiac remodeling. (a) Representative (a1) longitudinal and (a2) transverse H&E staining of left ventricle. Scale bar, 50 *μ*m. (b) Staining of Masson trichrome; dark blue indicates collagen fibers. Scale bar, 50 *μ*m. (c) Sirus red staining; note the dense collagen staining. Scale bar, 50 *μ*m. Immunohistochemical staining of (d) collagen I and (e) collagen III. Scale bar, 50 *μ*m. (f) Oil Red O staining quantifies lipid accumulation. Scale bar, 50 *μ*m. (g) The TUNEL assay was performed to identify DNA nicks; red plots indicate TUNEL-positive cells. Scale bar, 50 *μ*m. (h) Representative transmission electron micrographs. Scale bar, 2 *μ*m (h) Quantitative analysis of the cardiomyocyte size. (i) Semiquantification of collagen volume in total left ventricle. Data are presented as mean ± SD. ^∗∗^*P* < 0.01 CON vs. SHAM; ^##^*P* < 0.01 SG vs. SHAM. *n* = 6 in each group.

Structural and functional derangement of cardiac tissue is a key characteristic of DCM, consisting of hypertrophy, myocardial fibrosis, and contractile dysfunction in both diastolic and systolic phases. H&E staining showed hypertrophy of cardiomyocytes in the sham group, which was reversed after SG operation (Figures 3(a) and 3(i)). Sirius red and Masson trichrome staining detected a rising level of cardiac fibrosis in the sham group, whereas SG surgery brought about alleviation of fibrosis (Figures 3(b), 3(c), and 3(j)). Consistently, IHC staining of collagen I and collagen III showed elevated collagen deposition in the sham group, which was abrogated after SG operation (Figures 3(d) and 3(e)). Also, Oil Red O staining manifested those diabetic myocardial sections obviously stained with red droplets which were mitigated postoperatively, suggesting the alleviation of lipid deposition (Figure 3(f)). Furthermore, the TUNEL assay showed that SG reduced the ratio of TUNEL-positive cardiomyocytes indicative of decreased nicked DNA ends (Figure 3(g)). Transmission electron microscopy revealed disordered sarcomeres, swollen mitochondria with disorganized cristae, loss of intracellular contents, and the presence of pyroptosome in the heart of the sham group, whereas in the SG group, layers of uniformly shaped mitochondria with abundant and organized cristae intervened between regularly aligned myofibrils (Figure 3(h)). All of these results suggested that SG remarkably reversed the DCM-induced cardiac remodeling.

### 3.4. Effects of SG on NLRP3-Activated Pyroptosis in Cardiomyocytes

**Figure 4:**
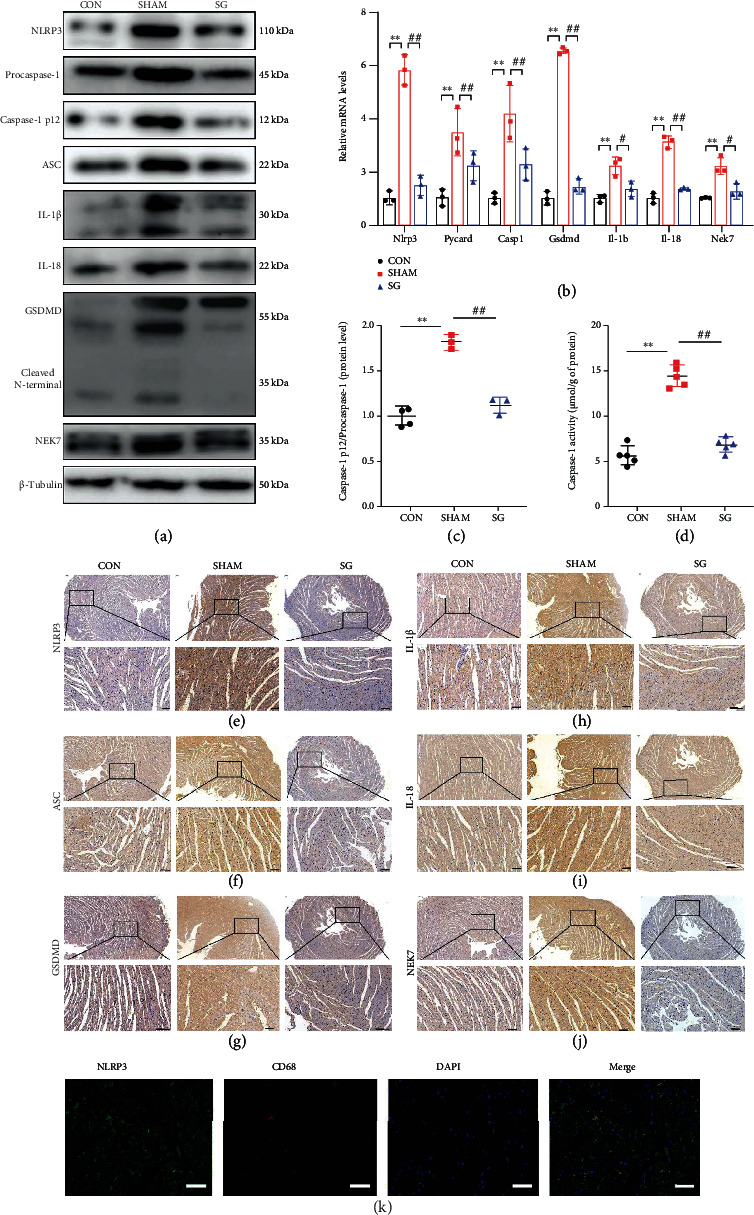
SG attenuates the activation of NLRP3 in diabetic myocardium. (a) Protein levels of NLRP3-related molecules shown by western blot. *β*-Tubulin was an internal reference control. *n* = 3 in each group. (b) Relative mRNA levels of *Nlrp3*-relaterd genes shown by real-time RT-PCR. *Tubb3* served as a reference gene. *n* = 3 in each group. (c) Ratio of caspase-1 p12/procaspase-1. *n* = 3 in each group. (d) Caspase-1 activity in heart tissues. *n* = 6 in each group. Immunohistochemical staining of (e) NLRP3, (f) ASC, (g) GSDMD, (h) IL-1*β*, (i) IL-18, and (j) NEK7. Scale bar, 50 *μ*m. (k) Double-labeling immunofluorescence of NLRP3 and CD68. Scale bar, 50 *μ*m. Data are presented as mean ± SD. ^∗∗^*P* < 0.01 CON vs. SHAM; ^##^*P* < 0.01 SG vs. SHAM. *n* = 3 in each group.

Pyroptosis serves as an inflammatory form of programmed cell death [10]. Previous studies have elucidated that the NLRP3 inflammasome is activated in the myocardium when exposed to hyperglycemia and pyroptosis which contributed to the development of DCM [13]. In this study, western blotting showed that NLRP3 inflammasomes were activated in the DCM model, as shown by the overexpression of NLRP3, apoptosis-associated speck-like protein (ASC), caspase-1, cleaved caspase-1, IL-1*β*, IL-18, NEK7, and GSDMD as well as its N-terminal fragment. Postoperatively, protein levels of the above related to the NLRP3-caspase 1 pathway were favorably reduced (Figure 4(a)). Similarly, real-time RT-PCR showed that relative transcriptional levels of NLRP3 inflammasome-related key genes were restrained in the SG group compared to the sham group (Figure 4(b)). Notably, a reluctantly high level of caspase-1 activity in the sham group was averted after SG performance (Figures 4(c) and 4(d)). Additionally, IHC staining of the three groups was performed to further confirm protein expression of NLRP3, ASC, GSDMD, and NEK7 (Figures 4(e)–4(g) and 4(j)). And the infiltration of inflammatory factors IL-1*β* and IL-18 was decreased post-SG surgery (Figures 4(h) and 4(i)). Using a macrophage marker CD68, we double labeled the NLRP3 and CD68 in heart sections and found that the NLRP3 was expressed not only in the macrophages but also in myocardial cells (Figure 4(k)). The above-mentioned results suggested that SG ameliorated the activation of NLRP3-related pathway molecules transcriptionally and posttranscriptionally in the diabetic myocardium.

### 3.5. High Glucose-Induced ROS Accumulation Acts as a Key Trigger of NLRP3-Mediated Pyroptosis in Cardiomyocytes

**Figure 5:**
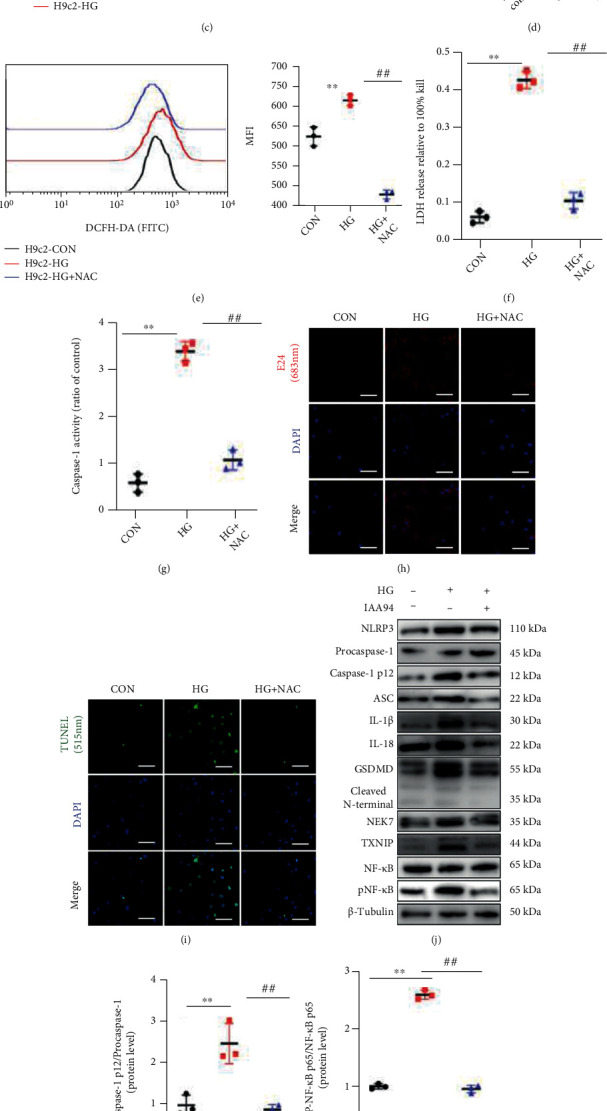
High glucose-induced ROS overload served as a trigger of NLRP3-mediated pyroptosis in H9c2 cells. (a) Protein expression of SOD2 in heart tissues. (b) mRNA expression of *Sod2* relative to *Tubb3* in hearts. (c) Flow cytometry quantified the 2-NBDG signals in HG-stimulated H9c2 cells. (d) Glucose concentrations in supernatants. (e) Intracellular ROS levels were assessed using DCFH-DA and flow cytometry. (f) The release of LDH percentage from H9c2 cells was reported to the total LDH release. (g) Caspase-1 activity of H9c2 cells. (h) H9c2 cells with membrane disintegrity were stained with red fluorescence by cell probe E42. (i) TUNEL staining of H9c2 cells. (j) Protein levels of NLRP3-related molecules shown by western blot in H9c2 cells treated with HG or HG+NAC. (k) The ratio of caspase-1 p12/procaspase-1 and (l) phospho-NF-*κ*B/total NF-*κ*B is shown. (m) Relative mRNA expression of *Nlrp3*-related molecules in H9c2 cells, *Tubb3* as the internal reference. Data are presented as mean ± SD. ^∗∗^*P* < 0.01 CON vs. HG; ^##^*P* < 0.01 HG+NAC vs. HG. *n* = 6 in 2-NBDG uptake experiment and *n* = 3 in each group in other experiments.

Hyperglycemia, associated with T2DM, increases the burden of oxidative stress [23]. Rising reactive oxygen species (ROS) production and impaired ROS clearance seem to contribute to the pathological progression of DCM [24]. It is well recognized that mitochondrial manganese superoxide dismutase (SOD2) played a role in ROS scavenging [25]. We found that the expression of SOD2 in the heart was lower in the sham group than in the SG group (Figures 5(a) and 5(b)). This finding suggested that the decline in ROS levels might have contributed to the restoration of cardiac dysfunction after SG surgery.

In order to mimic the in vivo environment of T2DM, H9c2 cells were starved in low-glucose DMEM with 2% FBS overnight, followed by stimulation with HG for 48 h. The ability of H9c2 cells to uptake the 2-NBDG was measured by flow cytometry. There was a decline in the mean fluorescence intensity (MFI) of 2-NBDG in HG-stimulated cells compared with control cells (Figure 5(c)). Additionally, glucose concentrations in the supernatant were determined by the GOD-POD method to indirectly reflect the ability of glucose uptake (Figure 5(d)). A marked reduction in the capacity for glucose uptake showed partial insulin resistance in HG-H9c2 cells. We pretreated cells for 12 h with NAC (1 mM), a specific ROS scavenger, to deplete intracellular ROS levels. After treatment, H9c2 cells were treated with the redox-sensitive fluorescent dye DCFH-DA for 0.5 h, and ROS levels were determined using flow cytometry. HG remarkably upregulated the fluorescent staining of ROS, whereas ROS levels were partially cleared by NAC (Figure 5(e)). Furthermore, we examined LDH release in culture supernatants and dyed membrane-disrupted cells with the probe E42 (Figures 5(f) and 5(h)). The outcomes of these two experiments suggested that high ROS accretion in the train of HG stimulation could arouse the plasma membranal disintegrity, which was a main feature in the pyroptosis of cells.

Caspase-1 activity, which is inevitable in NLRP3-dependent pyroptosis, zoomed in the HG condition and decreased with NAC treatment (Figure 5(g)). As the ROS burden rose with HG stimuli, there was a high proportion of TUNEL-positive cells of which DNA fragmentation might have been mediated by ROS and NLRP3 inflammasome-induced pyroptosis. However, the presence of NAC lowered the ratio of TUNEL staining (Figure 5(i)). Previous studies have demonstrated that the NLRP3 inflammasome is activated in a ROS/TXNIP/nuclear factor-*κ*B/p65 manner. Western blotting showed that HG upregulated the expression of NLRP3-related molecules, and this was alleviated by the ROS scavenger NAC (Figures 5(j)–5(l)). Real-time RT-PCR results were in agreement with the protein results (Figure 5(m)).

In summary, ROS might induce NLRP3 pathway-mediated pyroptosis in cardiomyocytes under the HG condition, which could be rescued by the ROS scavenger.

### 3.6. CLIC-Dependent Chloride Efflux Serves as an Upstream Event for Myocardial Pyroptosis Mediated by NLRP3 Inflammasome

**Figure 6:**
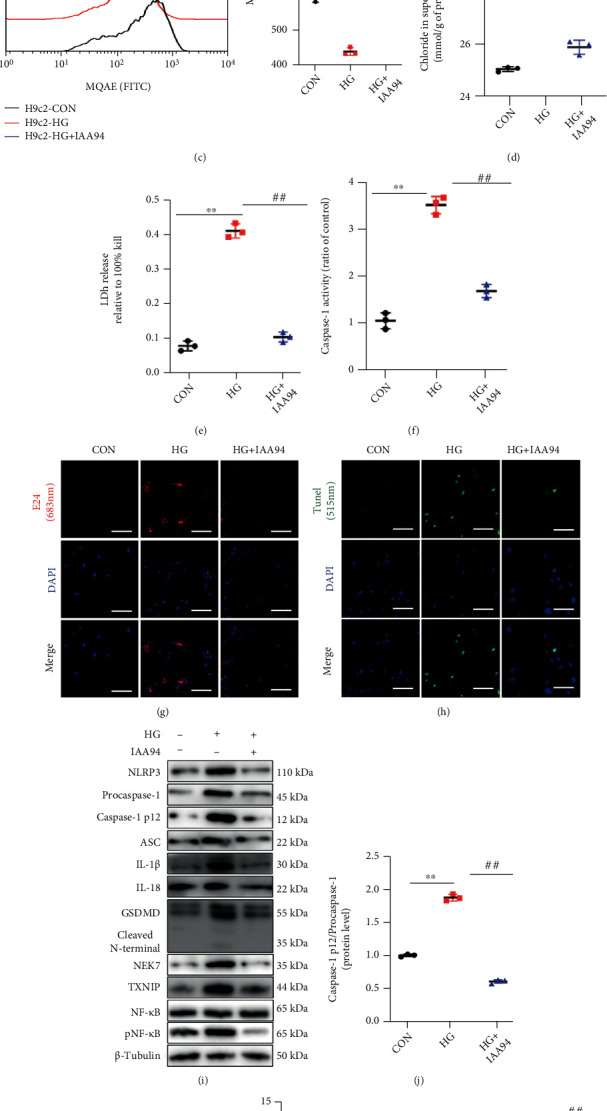
Chloride intracellular channel-dependent chloride efflux induced pyroptosis mediated by NLRP3 inflammasomes in H9c2 cells. (a) Protein expression of CLIC1 and CLIC4 in heart tissues. (b) Relative mRNA expression of *Clic1* and *Clic4* in hearts. (c) IAA-94 was used to block chloride efflux, and the intracellular chloride was measured with MQAE, a chloride-sensitive dye. (d) Chloride concentrations in supernatants. (e) The LDH release measured by a colorimetric assay. (f) Caspase-1 activity. (g) E42 staining indicated the membrane integrity. (h) TUNEL staining of H9c2 cells. (i) Protein levels of NLRP3-related molecules. (j) The ratio of caspase-1 p12/procaspase-1 and (k) the ratio of phospho-NF-*κ*B/total NF-*κ*B. (l) Relative mRNA expression of *Nlrp3*-related molecules in H9c2 cells, *Tubb3* as the internal reference. Data are presented as mean ± SD. ^∗∗^*P* < 0.01 CON vs. HG; ^##^*P* < 0.01 HG+IAA-94 vs. HG. *n* = 3 in each group.

The chloride efflux associated with the chloride intracellular channel (CLIC) proteins CLIC1 and CLIC4, were found to drive NEK7-NLRP3 activation [26]. Moreover, inhibition of volume-sensitive chloride currents had been reported to blunt apoptotic cell death and contractile dysfunction in cardiomyocytes [27]. Wondering whether chloride flow is involved in cardiac pyroptosis, we initially detected mRNA and protein levels of CLIC1 and CLIC4 in three rat models (Figures 6(a) and 6(b)). Both CLIC1 and CLIC4 declared a higher expression in DCM rats and decreased with the operation of SG. Identical HG stimulation was utilized for H9c2 cells, yet IAA-94 (100 *μ*M), which was a blocker of epithelial chloride channels, was used to inhibit the chloride efflux. The cells were loaded with the chloride-sensitive fluorescence probe MQAE to mark the intracellular chloride concentration via flow cytometry. The chloride efflux process was enhanced by an HG stimulus and could be abstained from by IAA-94 addition (Figure 6(c)). Measurement of chloride concentrations in supernatants by a colorimetric assay further confirmed this chloride concentration transition (Figure 6(d)). After chloride efflux inhibition, a reduction in LDH release and less dye with the probe E42 indicated repair of membrane disruption (Figures 6(e) and 6(g)). TUNEL staining and caspase-1 activity were also suppressed by IAA-94 (Figures 6(f) and 6(h)). Intriguingly, NLRP3-related molecules were also inhibited with the chloride efflux block, as shown by western blotting and real-time RT-PCR (Figures 6(i)–6(l)).

These findings suggest that intracellular chloride homeostasis functions as a signaling messenger to regulate NLRP3 inflammasome assembly and activation in cardiomyocytes.

### 3.7. ROS Might Function Upstream of Chloride Efflux and Promote Plasma Membrane Translocation of CLICs

**Figure 7:**
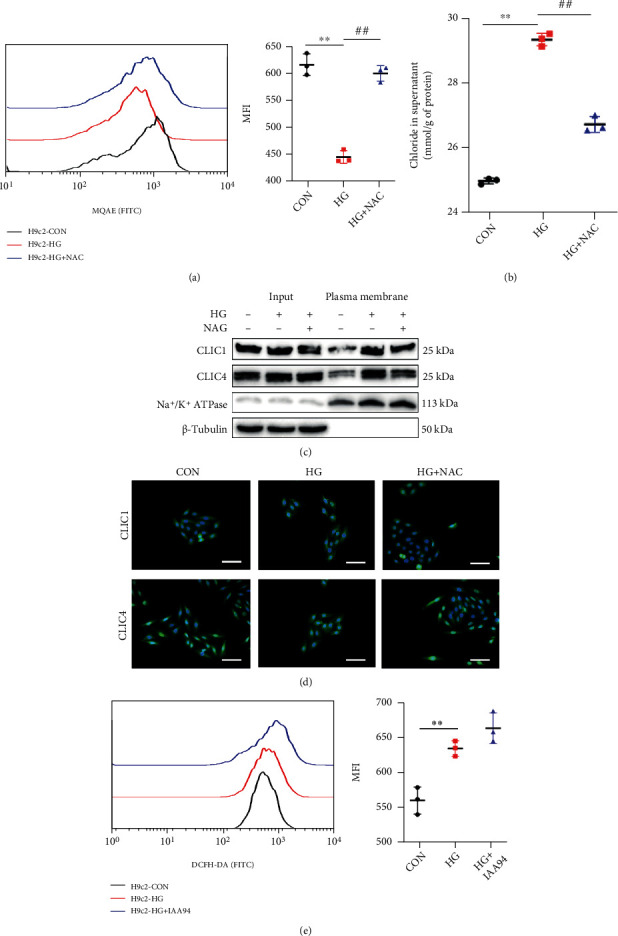
ROS promoted chloride efflux and plasma membrane translocation of CLICs in H9c2 cells. (a) The effect of ROS levels on chloride efflux was determined by MQAE in H9c2 cells with HG or HG+NAC treatments. (b) Chloride concentrations in supernatants after NAC treatment. (c) Immunoblot analysis of the indicated proteins in total lysates (input) or isolated plasma membrane in H9c2 cells treated with HG or HG+NAC. (d) Immunofluorescence of CLIC1 and CLIC4 in H9c2 cells. (e) ROS levels were assessed with DCFH-DA in H9c2 cells with HG or HG+IAA94 treatments. Data are presented as mean ± SD. ^∗∗^*P* < 0.01 CON vs. HG; ^##^*P* < 0.01 HG+IAA-94 vs. HG. *n* = 3 in each group.

Wondering if ROS promoted the CLIC-dependent chloride efflux in NLRP3 inflammasome activation in cardiac cells, we adopted the flow cytometry to examine chloride fluorescence intensity. We found that clearance of ROS production reversed the efflux of the chloride (Figure 7(a)). Chloride detection with a colorimetric assay also showed a decline in chloride concentrations in the supernatant (Figure 7(b)). Taken together, these findings suggested that chloride efflux was induced downstream of ROS production. Western analysis was conducted to detect membrane protein expression. Rearrangement of the CLIC structure with translocation to the cell membrane was observed in HG-induced high ROS conditions, while this was restored by NAC treatment (Figure 7(c)). Furthermore, plasma membrane translocation of CLIC1 and CLIC4 was shown with immunofluorescence (Figure 7(d)). Notwithstanding, provided that the chloride efflux was being inhibited by IAA-94, ROS levels were not altered afterwards (Figure 7(e)).

These results suggest that ROS can induce plasma membrane translocation of the CLIC-induced chloride efflux in cardiomyocytes, ultimately leading to NLRP3 activation.

## 4. Discussion

Bariatric surgery has remarkable metabolic benefits in obesity, T2DM, and following complications [6, 28, 29]. In the present study, we found that SG surgery alleviated the capability of myocardial contraction and diastole and delayed the progression of DCM. The deactivation of NLRP3 inflammasome-mediated myocardial pyroptosis was observed after SG *in vivo*. As found *in vitro*, we speculate that the ROS–chloride efflux axis might perform as an upstream trigger for NLRP3 inflammasome activation in myocytes.

In the present study, we adopted the method of HFD feeding combined with low-dose STZ injection intraperitoneally to establish the DCM model. This procedure is widely acknowledged by researchers worldwide because it simulates the natural history and metabolic features of T2DM [30, 31]. Additionally, this method has been confirmed by previous studies on the induction of DCM models [31, 32]. In line with previous researches [33, 34], metabolic benefits of bariatric surgery were observed in our study, characterized by a reduced body weight, food intake, and blood glucose levels and restored glucose tolerance as well as insulin sensitivity postoperatively. These improvements guaranteed the effect of SG surgery (Figure 1).

DCM is initially characterized by impaired cardiac insulin metabolic signaling with altered diastolic relaxation [35, 36]. Therefore, echocardiography was used to identify cardiac function in this study, and impairment of diastolic and systolic function in rats with DCM was improved after SG surgery. But the cardiac function cannot be restored as good as normal control rats. Notably, a well-recognized indicator of insulin sensitivity is glucose uptake [37]. ^18^F-FDG-labelled PET-CT scans were carried out to visualize the ability of glucose uptake. We observed a marked decline in the glucose uptake in rats with DCM, and this was restored after SG surgery. These results consistently showed the potential therapeutic value of bariatric surgery in DCM [9, 38] (Figure 2).

Diabetic cardiac dysfunction exists due to pathological changes in the heart, such as cardiac hypertrophy, fibrotic myocardium remodeling, cell death, and ectopic lipid deposition [39–42]. In this study, cardiac hypertrophy was alleviated after SG surgery. Collagen deposition and fibrosis contribute to impaired contractility [42]. The excessive collagen distribution in both the interstitial and perivascular areas was remarkably diminished after the SG operation. Lipid deposition was also reduced with SG, which could lead to relief of lipotoxic cardiomyopathy [43]. These findings suggest that functional improvement was obtained by SG, ascribing these benefits to morphological transformation.

Pyroptosis is a type of programmed inflammatory cell death, which is provoked by sensing of danger or pathogenic signals and characterized by DNA damage, cell swelling, lysis, and inflammatory responses [44, 45]. Pyroptotic cells can also lead to DNA damage and become positive with TUNEL staining [46]. The NLRP3 inflammasome is a typical macromolecular trigger of pyroptosis. This inflammasome is a multimeric complex assembly of adapter and effector proteins, consisting of NLRP3, adapter protein apoptosis-associated speck-like protein, and caspase-1 [47]. The NLRP3 inflammasome serves as a platform to engage procaspase-1, which come into active ones through oligomerization. Activated caspase-1 then processes pro-IL-1*β* and pro-IL-18 to generate their active forms [48]. Additionally, the NLRP3 assembly primes the cleavage of pore-forming gasdermin D protein to initiate pyroptosis [49]. Recent evidence has shown a new component of the inflammasome, NEK7, which is a serine-threonine kinase associated with mitosis. NEK7 binds to NLRP3 to trigger its assembly [50]. In this study, we found more TUNEL-positive cells in the sham group than in the SG group. Additionally, SG treatment normalized alterations in myofilaments, swollen mitochondria, and the decreased number of cristae in diabetic rats (Figure 3). In this study, NLRP3-related molecules were activated in the diabetic myocardium, while they were inhibited with SG surgery. Taken together, we confirmed that SG surgery indeed alleviated the NLRP3 inflammasome activation and reversed myocardial pyroptosis (Figure 4). We hypothesize that the NLRP3 inflammasome is a pivotal reason why SG defers or even reverses the progression of DCM.

Abundant evidence has verified that ROS is important in the pathogenesis of T2DM [51]. ROS overload has been proposed as a mechanism involved in activating the NLRP3 inflammasome [52]. ROS-induced NF-*κ*B activation during metabolic reprogramming incurs rapid NLRP3 transcription, thereby facilitating the NLRP3 inflammasome overexpression and the maturation of caspase-1 and IL-1b [53]. In regard to TXNIP, it is a ROS-sensitive molecule implicated in T2DM and a key regulator of NLRP3 inflammasome activation [54, 55]. In the current study, ROS levels were abrogated by SG. Additionally, we found that HG-stimulated H9c2 cardiomyocytes were under the condition of membrane disintegrity and DNA fraction, which implied myocardial pyroptosis. The NLRP3 pathway was also activated in the HG condition, in line with previous studies [13]. Use of a ROS scavenger led to a remarkable reduction in high NLRP3 expression. These results suggested that ROS acted as a crucial regulator of NLRP3 inflammasomes in myocytes (Figure 5).

Recent research has shown that the chloride efflux acts downstream of mitochondrial ROS production to activate the NLRP3 inflammasome in macrophages [26]. Additionally, inhibition of volume-sensitive chloride currents reduces cell death and reverses contractile dysfunction in cardiomyopathies [27]. To determine the mechanism participating in NLRP3 inflammasome activation in myocytes, we initially confirmed the transcriptional and posttranscriptional downregulation of CLICs after SG surgery. Using a chloride channel blocker, we observed restored myocardial NLRP3-mediated pyroptosis under a HG stimulus. This finding indicated that the chloride efflux might act as a signaling messenger to regulate NLRP3 assembly and activation directly or indirectly (Figure 6). Furthermore, we implied in our study that the chloride efflux acted downstream of ROS generation and that oxidative stress promoted CLICs to translocate to the cell membrane for biological function (Figure 7).

This study has several limitations. Firstly, the two general effects of SG are the weight loss and decrease in blood glucose, which both contribute to the amelioration of DCM development. So, it is tough to define whether the SG could directly alleviate DCM or the SG's outcome played the pivotal role. What is more, experiments will be further required to directly associate SG and the alleviation of DCM with the role of ROS-chloride efflux-mediated NLRP3 inflammasome as an underlying mechanism.

Taken together, our study provides evidence that the NLRP3 inflammasome is downregulated in DCM after the SG operation. Additionally, the ROS overburden–chloride efflux axis might act as the proximal event before NLRP3 activation in the myocardium, thus contributing to morphological and functional alleviation of DCM. Notwithstanding, whether alleviation of NLRP3 inflammasome and its upstream mechanisms in the SG operation could be used as a new therapeutic target for T2DM and its related comorbidities remains to be further explored and elucidated.

## 5. Conclusions

Collectively, we identified for the first time that alleviation of NLRP3 inflammasome dysregulation and its upstream mechanisms by SG surgery, including ROS overload and chloride efflux, might embrace a rosy future as a promising therapeutic strategy for diabetes mellitus and its related comorbidities.

## Figures and Tables

**Table 1:** Primers used for qRT-PCR.

Gene	Sequence
*Nlrp3*	Forward: 5′-GCAGCGATCAACAGGCGAGAC-3′ Reverse: 5′-TCCCAGCAAACCTATCCACTCCTC-3′
*Pycard*	Forward: 5′-GATTATGGAAGAGTCTGGAGCTGTGG-3′ Reverse: 5′-ATGAGTGCTTGCCTGTGTTGGTC-3′
*Casp1*	Forward: 5′-AAACACCCACTCGTACACGTCTTG-3′ Reverse: 5′-AGGTCAACATCAGCTCCGACTCTC-3′
*Gsdmd*	Forward: 5′-AAGACTTCCAAGGCCTGCGT-3′ Reverse: 5′-CATGCTGGGCTGGTCCTGTA-3′
*Il-1b*	Forward: 5′-GACCTGTTCTTTGAGGCTGACA-3′ Reverse: 5′-CTCATCTGGACAGCCCAAGTC-3′
*Il-18*	Forward: 5′-CGACCGAACAGCCAACGAATCC-3′ Reverse: 5′-TCACAGATAGGGTCACAGCCAGTC-3′
*Nek7*	Forward: 5′-GGCTGTCTGCTGTATGAGATGGC-3′ Reverse: 5′-GATCTGATGGGAGAGGTGGGTAGTC-3′
*Txnip*	Forward: 5′-GCCAGACCAAAGTGCTCACTCAG-3′ Reverse: 5′-GAGACTCTTGCCACGCCATGATG-3′
*Sod2*	Forward: 5′-TCCCTGACCTGCCTTACGACTATG-3′ Reverse: 5′-TCGTGGTACTTCTCCTCGGTGAC-3′
*Clic1*	Forward: 5′-TCCCAGCCATAAACCATCCATTGTG-3′ Reverse: 5′-ATTCTGCCAGGGTGCTTTCTCTTG-3′
*Clic4*	Forward: 5′-TGAACGGACTGAAGGAGGAGGAC-3′ Reverse: 5′-GGTGGTGACACTGAACACGACTC-3′
*Tubb3*	Forward: 5′-CGTCCACCTTCATCGGCAACAG-3′ Reverse: 5′-TCGGCCTCGGTGAACTCCATC-3′

## Data Availability

The detailed data used to support the findings of this study are available from the corresponding author upon request.
